# Reduced skeletal muscle oxidative capacity and impaired training adaptations in heart failure

**DOI:** 10.14814/phy2.12353

**Published:** 2015-04-08

**Authors:** William M Southern, Terence E Ryan, Kirsten Kepple, Jonathan R Murrow, Kent R Nilsson, Kevin K McCully

**Affiliations:** 1 University of GeorgiaAthens, Georgia; 2 East Carolina UniversityGreenville, North Carolina; 3 Georgia Regents UniversityAthens, Georgia

**Keywords:** Exercise training, heart failure, mitochondria, near-infrared spectroscopy, skeletal muscle

## Abstract

Systolic heart failure (HF) is associated with exercise intolerance that has been attributed, in part, to skeletal muscle dysfunction. The purpose of this study was to compare skeletal muscle oxidative capacity and training-induced changes in oxidative capacity in participants with and without HF. Participants with HF (*n* = 16, 65 ± 6.6 years) were compared with control participants without HF (*n* = 23, 61 ± 5.0 years). A subset of participants (HF: *n* = 7, controls: *n* = 5) performed 4 weeks of wrist-flexor exercise training. Skeletal muscle oxidative capacity was determined from the recovery kinetics of muscle oxygen consumption measured by near-infrared spectroscopy (NIRS) following a brief bout of wrist-flexor exercise. Oxidative capacity, prior to exercise training, was significantly lower in the HF participants in both the dominant (1.31 ± 0.30 min^−1^ vs. 1.59 ± 0.25 min^−1^, *P* = 0.002; HF and control groups, respectively) and nondominant arms (1.29 ± 0.24 min^−1^ vs. 1.46 ± 0.23 min^−1^, *P* = 0.04; HF and control groups, respectively). Following 4 weeks of endurance training, there was a significant difference in the training response between HF and controls, as the difference in oxidative training adaptations was 0.69 ± 0.12 min^−1^ (*P* < 0.001, 95% CI 0.43, 0.96). The wrist-flexor training induced a ∼50% improvement in oxidative capacity in participants without HF (mean difference from baseline = 0.66 ± 0.09 min^−1^, *P* < 0.001, 95% CI 0.33, 0.98), whereas participants with HF showed no improvement in oxidative capacity (mean difference from baseline = −0.04 ± 0.08 min^−1^, *P* = 0.66, 95% CI −0.24, 0.31), suggesting impairments in mitochondrial biogenesis. In conclusion, participants with HF had reduced oxidative capacity and impaired oxidative adaptations to endurance exercise compared to controls.

## Introduction

Heart failure (HF) is a widespread condition in the United States, affecting more than 6 million individuals (Roger et al. 2012), and is often associated with multiple comorbidities such as hypertension, diabetes, and hypercholesterolemia (Braunstein et al. 2003; van Deursen et al. 2014). A common symptom of HF is exercise intolerance, which, interestingly, is poorly correlated with objective measures of cardiac function (Franciosa et al. 1981). This discrepancy between cardiac function and exertional fatigue can be explained by peripheral skeletal muscle and vascular abnormalities. Skeletal muscle wasting and compositional changes (Anker et al. 1997; Duscha et al. 1999; Fulster et al. 2013; Haykowsky et al. 2013, 2014), endothelial dysfunction (Treasure et al. 1990; Kubo et al. 1991; Drexler et al. 1992a), shift in fiber type from oxidative to glycolytic (Mancini et al. 1989; Sullivan et al. 1990; Simonini et al. 1996a; Kitzman et al. 2014), and reduced metabolism (Massie et al. 1988; Mancini et al. 1989; Okita et al. 1998; Abozguia et al. 2008) have all been identified as skeletal muscle and vascular abnormalities associated with HF. In addition, reduced skeletal muscle oxidative capacity has also been proposed as a muscle abnormality associated with HF as multiple human and rodent studies have shown reductions in skeletal muscle mitochondria function, density, size, and mitochondrial enzymes (e.g., citrate synthase and succinate dehydrogenase) (Sullivan et al. 1990; Arnolda et al. 1991; Drexler et al. 1992b; Mancini et al. 1992; Simonini et al. 1996a,b; Delp et al. 1997; Garnier et al. 2003; Schrepper et al. 2012). While there is clearly a HF-induced skeletal muscle myopathy contributing to exercise intolerance, some research has suggested that mitochondrial dysfunction could be the product of muscle disuse and deconditioning as a result of a diminished capacity to exercise rather than a HF-induced myopathy (Chati et al. 1996; Mettauer et al. 2001; Williams et al. 2004; Toth et al. 2012).

In addition to determining how skeletal muscle mitochondria are involved in a HF-related myopathy, it is important to understand how they might respond to exercise training. While exercise has been shown to yield muscular oxidative improvements in people with HF (Minotti et al. 1990; Hambrecht et al. 1995; Wisloff et al. 2007), the capacity for improvement has not been assessed. In fact, to our knowledge, no study has directly compared the magnitude of oxidative adaptations to endurance exercise training between participants with and without HF. Exercise training has been recommended for individuals with HF as a safe and effective means of improving clinical, functional, and physiological outcomes (O'Connor et al. 2009; Keteyian 2011; McMurray et al. 2012; Ades et al. 2013), thus, it is important to understand how muscle mitochondria in individuals with HF respond to exercise training.

The first aim of this study was to measure and compare baseline skeletal muscle oxidative capacity of people with and without HF. The second aim of this study was to compare the magnitude of oxidative adaptations in response to endurance exercise in people with and without HF. We hypothesized that people with HF would have reduced oxidative capacity and impaired oxidative adaptations to endurance exercise compared to controls.

## Materials and Methods

### Participants

Sixteen participants with HF (13 male, 3 female) were recruited through a local cardiology clinic. Twenty healthy controls (6 male, 17 female) were recruited from the surrounding community. A subset of enrolled participants (*n* = 7, *n* = 5; for HF and control respectively) volunteered to participate in a 4-week long wrist-flexor training program, followed by 4 weeks of inactivity. One HF participant only completed the first 5 weeks of the program. One control participant completed the first 7 weeks of the program.

HF participants with implanted cardioverter-defibrillators (ICDs) and reduced ejection fraction (<35%) were recruited for the study. All corresponding clinical data for the HF participants were obtained from Athens Regional Cardiology with permission from the HF participants. Individuals with HF were excluded from participation in the study if they had stable HF for less than 3 months, their initial ejection fraction was above 35%, or if they were considered New York Heart Association Functional Class I or IV. General exclusion criteria for both groups included uncontrolled Type-I or Type-II diabetes, individuals who currently smoke, and those currently engaged in wrist-flexor training. This study was conducted with the approval of the Institutional Review Board at the University of Georgia (Athens, GA) and at Athens Regional Medical Center. All participants gave written, informed consent before being enrolled in the study.

### Experimental design

Wrist-flexor muscles were selected because they are not involved in locomotion, and therefore should be untrained and independent of habitual physical activity levels compared with other muscles of the lower body such as the calf or thigh. Near-infrared spectroscopy (NIRS) was used to assess skeletal muscle oxidative capacity as previously reported (Ryan et al. 2012). This study included both a cross-sectional and an intervention component. The cross-sectional component consisted of a single testing session, in which measurements of skeletal muscle oxidative capacity were made on both the dominant (DOM) and nondominant (nDOM) arms (one HF participant was under a time constraint and only one arm was measured). The intervention component consisted of a 4-week wrist-flexor endurance exercise training program, followed by 4 weeks of detraining. The training was performed on the nDOM arm, which was considered to be the least active of the two arms, whereas the DOM served as the nontraining control. Skeletal muscle oxidative capacity was measured every 7–8 days on both the DOM and nDOM arms.

### Exercise training

The wrist-flexor training consisted of 16 total training sessions (4 days per week, 30 min per day for 4 weeks) on the nDOM arm only. During the first training session, participants were given a hand weight set to approximately 15% of their MVIC and a tolerable training intensity (i.e., contraction frequency) was determined for each participant. Every week, the contraction frequency was increased as tolerated by each participant. One supervised training session was performed each week following measurements of oxidative capacity. Each participant was then sent home with a fixed metronome and hand weight in order to perform the remaining three training sessions at his or her home. After completing each home-based training session, each participant was asked to provide a report of the training to the study supervisor. After the 16 training sessions were completed, participants were encouraged to avoid any wrist-flexor training for the remainder of the study.

### NIRS measurements of skeletal muscle oxidative capacity

Experimental procedures in this study were similar to a previous study in our lab (Ryan et al. 2013a). Briefly, participants were asked to lie supine on a padded table, with the arm to be tested extended 90° from their body. The NIRS device was placed directly over the wrist-flexor (i.e., palmaris longus, flexor carpi ulnaris, and flexor carpi radialis) musculature. NIRS signals were obtained using a continuous-wave NIRS device (Oxymon MK III, Artinis Medical Systems, The Netherlands). The NIRS signals provided by the device included: oxygenated hemoglobin (O_2_Hb), deoxygenated hemoglobin (HHb), Hb_difference_ (Hb_difference_ = O_2_Hb – HHb), and total blood volume (tHb) (tHb = O_2_Hb + HHb). Resting blood flow measurements were measured as the rate of increase in the tHb signal during three 10 sec venous occlusions (∼65–70 mmHg) (Van Beekvelt et al. 2001). Resting muscle oxygen consumption (resting mVO_2_) was measured as the rate of decrease in Hb_difference_ during two 30 sec arterial occlusions (∼250–275 mmHg) (Ryan et al. 2012). To normalize the NIRS signals, an ischemic/hyperemia calibration (i.e., ∼3–5 min arterial occlusion) was performed as previously described (McCully et al. 1994; Southern et al. 2014).

To measure the oxidative capacity, a short duration exercise was used increase skeletal muscle oxygen consumption followed by repeated arterial occlusions to measure the rate of recovery of mVO_2_ as previously described (Motobe et al. 2004; Hamaoka et al. 2007; Buchheit et al. 2011; Ryan et al. 2012). The series of arterial occlusions were performed in the following manner: occlusions 1–5 (3–5 sec on/3–5 sec off), occlusions 5–10 (7 sec on/7 sec off), occlusions 10–15 (10 sec on/10 sec off), and occlusions 15- (10 sec on/20 sec off). The post-exercise mVO_2_ measurements were fit to a mono-exponential curve as previously described (Ryan et al. 2012). The rate constant (k) for the recovery of mVO_2_ is directly related to the muscle's maximal oxidative capacity (Ryan et al. 2013b, 2014).

### Strength testing

To determine each participant's wrist-flexor training weight and to track any changes in wrist-flexor strength during the training protocol, maximal voluntary isometric contractions (MVIC) were performed each week using a either a JAMAR® handgrip dynamometer (Sammons Preston Rolyan, Bolingbrook, IL) or a home-built handgrip dynamometer using a Rice Lake force transducer (Rice Lake Weighing Systems, Rice Lake, WI), BIOPAC© device and AcqKnowledge® software. Participants were given a calibrated hand dynamometer and instructed to perform three consecutive MVICs. Participants were allowed to rest for 20 sec between each contraction. The average value of the three contractions was recorded and used for the final analysis.

### Measurement of physical activity

Physical activity was assessed in each group using the International Physical Activity Questionnaire (IPAQ) long form. The total amount of activity per week was calculated according to the “Guidelines for the Data Processing and Analysis of the “International Physical Activity Questionnaire” (Karolinska Institutet, Sweden) and used for analysis.

### Statistical analysis

Data are presented as means ± SD. Statistical analyses were performed using SPSS 19.0 (IBM®, Armonk, NY). Unpaired *t*-tests were used to compare between groups for resting mVO_2_, blood flow, and baseline oxidative capacity. Analysis of the contraction frequency, oxidative capacity, and strength measurements of the intervention component of the study consisted of a two-way (time × group) mixed model repeated measures ANOVA with a within subjects factor of time and a between subject factors of group. If a significant interaction was found, post hoc analysis with a Bonferroni correction was performed on the main effect. Significance was accepted at *P* < 0.05.

## Results

One participant in the HF cohort only completed three training sessions due to inflammation and arthritis in the hand. This participant's data were only included in the cross-sectional component of the study. Table1 displays the baseline participant characteristics for both the cross-sectional and training studies.

**Table 1:** Participant characteristics

All participants	HF (*n* = 16)	Control (*n* = 23)
Age (years)	65 ± 6.6	61 ± 4.9
Sex (M/F)	13/3	6/17
Height (cm)*	180.7 ± 8.5	169.0 ± 8.4
Weight (kg)*	98.0 ± 13.1	78.4 ± 20.0
PA (met-min week^−1^)	1804 ± 1944	3132 ± 2963
DOM ATT (cm)	0.67 ± 0.3	0.79 ± 0.3
nDOM ATT (cm)	0.68 ± 0.2	0.81 ± 0.4
Length of HF (years)	10.6 ± 10.6	–
Etiology		
Ischemic	13	–
Non-Ischemic	3	–
Ejection fraction (%)	29 ± 11.5	–
Beta-blockers	13	0
ACE inhibitors	10	0
Digoxin	5	0
Statins	14	0
Metformin	5	0

Training participants	HF (*n* = 7)	Control (*n* = 5)
Age (years)	66 ± 4.1	61 ± 5.5
Sex (male/female)	6/1	2/3
Height (cm)	184.0 ± 3.6	172.2 ± 12.2
Weight (kg)	101.8 ± 10.6	76.7 ± 20.9
PA (met-min week^−1^)	1490 ± 2363	1896 ± 2155
DOM ATT (cm)	0.60 ± 0.3	0.66 ± 0.3
nDOM ATT (cm)	0.71 ± 0.3	0.61 ± 0.2
Beta-blockers	5	0
Statins	6	0
Metformin	2	0

Note: Data are presented as mean ± SD; PA = physical activity, PA was collected on 14 of the 15 HF participants and 24 of the 26 controls; DOM = dominant arm; nDOM = nondominant arm; ATT = adipose tissue thickness; ACE = angiotensin-converting enzyme; ^*^Indicates significance at *P* < 0.05.

### Exercise training

**Figure 1:**
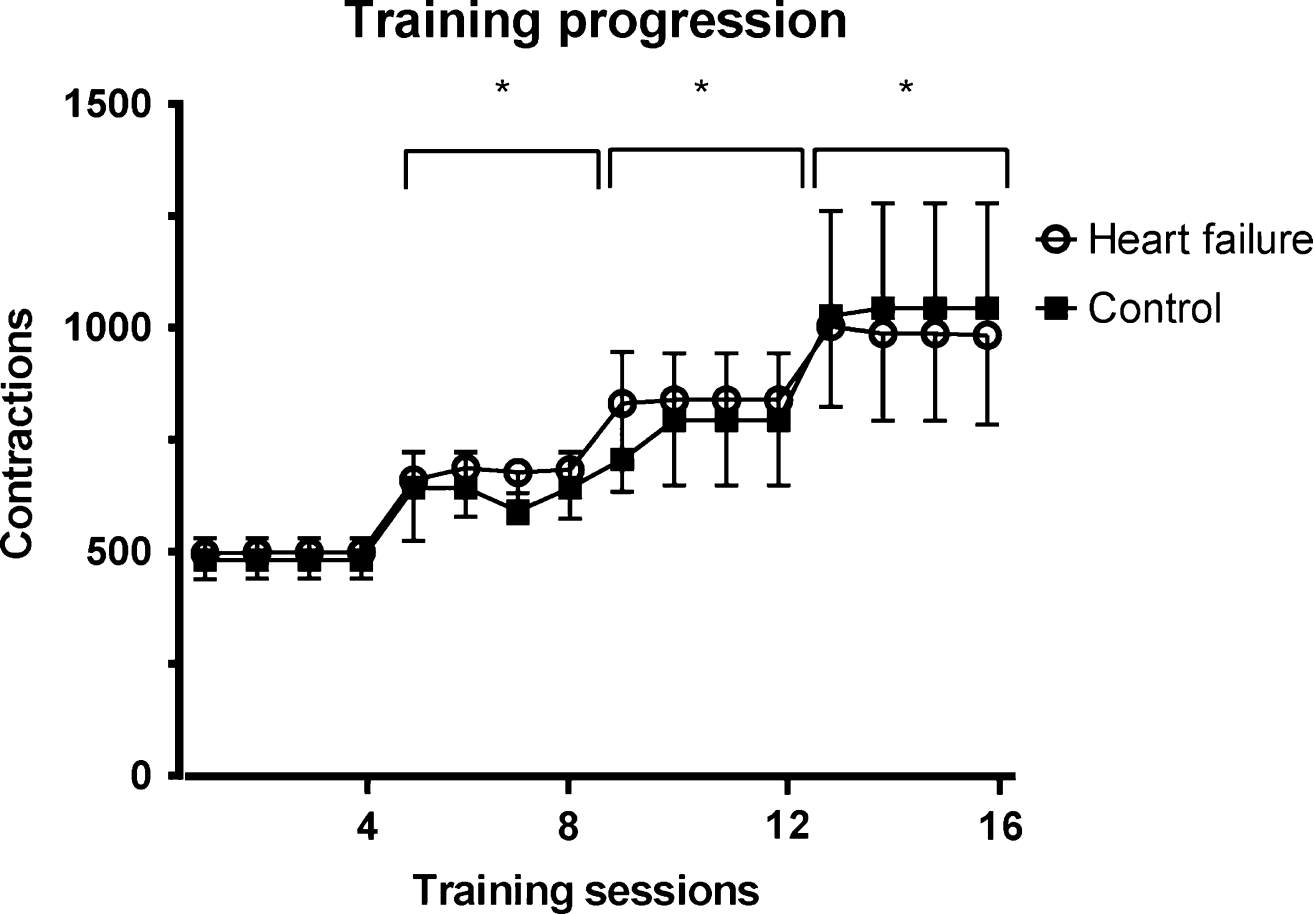
Progression of wrist-flexor contractions performed during each 30-min training session. The training progression was identical for both heart failure (open circles) and control groups (solid squares). Frequency of wrist-flexor contractions was increased each week, but the duration of training sessions remained at 30 min. *P < 0.001 different from week 1. No differences were found between groups at any training level (*P* = 0.13). Data are presented as mean ± SD.

Adherence to the home-based training was very high in both groups. In both groups, all participants reported completing the entire 30 min of wrist-flexor training for all 16 sessions, with the exception of two participants in the control group who reported completing only 20 of the 30 min for one training session each. Training volume was increased each week, as all participants increased wrist-flexion contraction frequency each week (*P* < 0.001) (Fig.1). No difference was found in the contraction frequency between HF and controls at any point during the training (*P* = 0.125).

### Resting measurements

**Figure 2:**
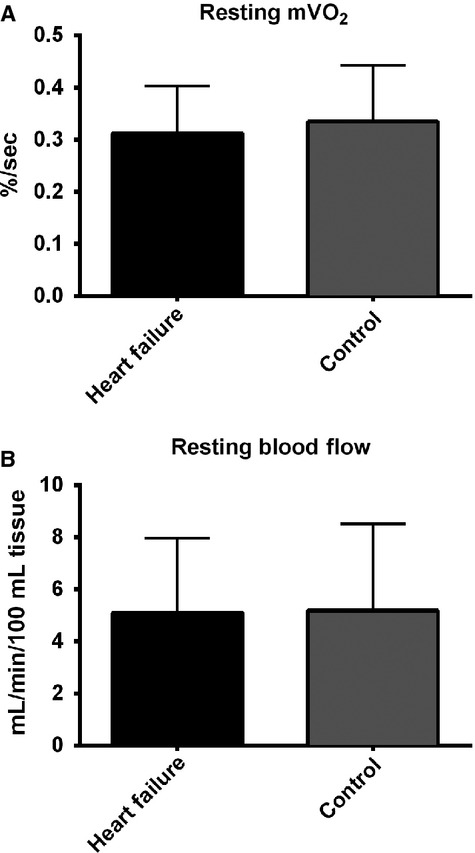
(A) Resting muscle oxygen consumption (mVO_2_) in wrist-flexors of HF and control groups. (B) Resting blood flow in wrist-flexors of HF and control groups. Each dataset represents the combined mean from both arms. *P < 0.005 for difference between HF and control.

No difference in resting mVO_2_ was found between HF and controls (0.31 ± 0.09 vs. 0.33 ± 0.11% per sec, for HF and control, respectively, *P* = 0.23) (Fig.2A). Resting blood flow was not different between HF (5.1 ± 2.9 mL min^−1^ 100 mL^−1^) and controls (5.2 ± 3.3 mL min^−1^100 mL^−1^, *p =* 0.63) (Fig.2B). Baseline strength for the control group was 28.1 ± 17.1 kg and 25.0 ± 15.1 kg for the DOM and nDOM arms, respectively. Baseline strength for the HF group was 27.6 ± 5.4 kg and 25.7 ± 3.8 kg for the DOM and nDOM arms, respectively. There was no difference in strength between HF and controls, and strength did not change during the course of the training intervention for either group.

### NIRS measurements of skeletal muscle oxidative capacity

**Figure 3:**
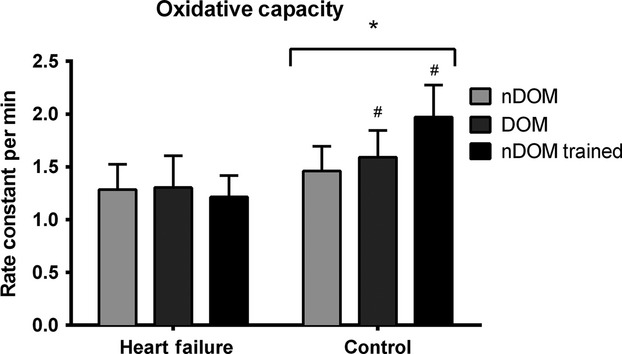
Skeletal muscle oxidative capacity of both heart failure and control groups for nondominant arm (nDOM) at baseline, dominant arm (DOM) at baseline, and nondominant arm after 4 weeks of wrist-flexor training (nDOM trained). Data are expressed as rate constants for the postexercise recovery of mVO_2_. *P < 0.05 for different from HF; ^#^*P* < 0.05 for different from nDOM arm.

Oxidative capacity in the DOM arm was significantly reduced in HF compared to controls (1.31 ± 0.30 min^−1^ vs. 1.59 ± 0.25 min^−1^, *P =* 0.002). Oxidative capacity was also significantly reduced in the nDOM arm in HF compared to controls (1.29 ± 0.24 min^−1^ vs. 1.46 ± 0.23 min^−1^, *P =* 0.04). There was no difference between the DOM and nDOM in the HF group (1.33 ± 0.30 min^−1^ vs. 1.29 ± 0.24 min^−1^, *P =* 0.40). However, in the control group, the DOM arm had higher oxidative capacity than the nDOM arm (1.59 ± 0.25 min^−1^ vs. 1.46 ± 0.23 min^−1^, *P =* 0.03). The oxidative capacity data for each arm and each group are displayed in Fig.3.

**Figure 4:**
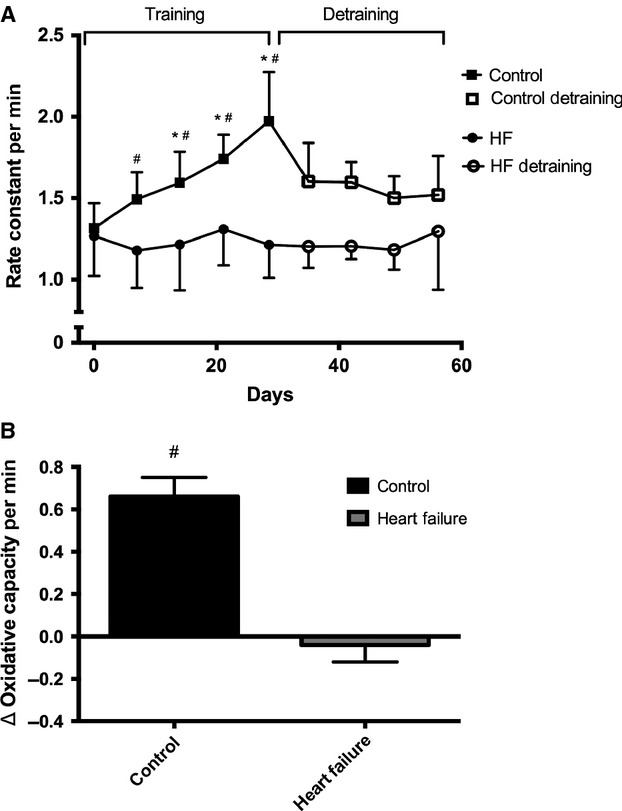
(A) Change in oxidative capacity expressed as rate constants for the postexercise recovery of mVO_2_ for heart failure (circles) and controls (squares) during 4 weeks of wrist-flexor exercise training (solid markers) and detraining (open markers). (B) Mean change in oxidative capacity from baseline following 4 weeks of wrist-flexor exercise training for heart failure (gray bar) and controls (black bar). Data presented as mean ± SD. *P < 0.05 for difference from day 0; ^#^*P* < 0.05 for different from HF.

Among the participants who engaged in wrist-flexor training, the difference in the training responses between the HF and control groups was 0.69 ± 0.12 min^−1^ (*P* < 0.001, 95% CI 0.43, 0.96, Fig.4B), revealing a significant difference in the training adaptations between the HF and control groups. Further within group comparisons revealed that wrist-flexor exercise training induced a ∼50% improvement in oxidative capacity from baseline to week four of training in the control group (mean difference from baseline within control group* =* 0.66 ± 0.09 min^−1^, *P* < 0.001, 95% CI 0.33, 0.98, Fig.4A). In contrast, in the HF group, a ∼2% decrease in oxidative capacity was found from baseline to week 4 of training (mean difference from baseline within HF group* =* −0.04 ± 0.08 min^−1^, *P =* 0.66, 95% CI −0.24, 0.31, Fig.4A).

Between arm analysis (DOM vs. nDOM) was performed for both the HF and control training cohorts. There was a significant difference between arms over time only in the control group [*F*(4, 32) = 4.47, *P *< 0.01]. The HF group displayed no indication of a training effect [*F*(4, 48) = 0.76, *P* = 0.56].

## Discussion

The results of this study indicate that oxidative capacity was reduced in participants with HF compared to controls. Our results are consistent with previous research that has reported reduced oxidative capacity in participants with HF (Drexler et al. 1992b; Mancini et al. 1992). Furthermore, we found that a subset of participants from the HF group did not show an improvement in oxidative capacity after 4 weeks of exercise training. Interestingly, these results do not agree with previous studies that have reported improvements in oxidative capacity in people with HF (Minotti et al. 1990; Hambrecht et al. 1995; Belardinelli et al. 1999). In contrast, a subset of control participants demonstrated a linear increase in oxidative capacity, with an average improvement of ∼50% by the end of training, a result similar to that for a young healthy population (Ryan et al. 2013a). The improvements elicited by the control group have also been supported by numerous studies that have documented similar training adaptations in oxidative capacity using in vivo and in vitro methodologies (Gollnick et al. 1973; Henriksson 1977).

The cross-sectional portion of this study revealed that oxidative capacity was reduced (∼21% and ∼13% in DOM and nDOM arms, respectively) in the skeletal muscle of HF participants compared to controls. Interestingly, there has not been universal agreement in the literature regarding reduced skeletal muscle oxidative capacity in HF. While several studies have reported diminished markers of oxidative capacity in HF (Sullivan et al. 1990; Drexler et al. 1992b; Schaufelberger et al. 1997), these results may reflect a lack of experimental control for physical activity. For example, Sullivan et al. (1990) found reduced percentage of type I fibers and oxidative enzyme capacity in participants with HF compared to controls, but reported over half of the control group was active and engaging in regular exercise. Similarly, Schaufelberger et al. (1997) found comparable skeletal muscle alterations, but did not report physical activity or fitness level of controls. In contrast, Mettauer et al. recruited controls that were unfit and physically inactive (peak VO_2_ ∼30 mL kg^−1^ min^−1^), and the authors found that mitochondrial function, as assessed by in situ measurements of maximal ADP-stimulated respiration rates, was not different between HF and controls. In addition, Toth et al. (2012) matched and monitored physical activity between HF and controls using accelerometry, and reported no difference in oxidative enzymes between the groups, but did find a trend toward fewer mitochondria per muscle fiber. In this study, the whole body physical activity of the controls was not matched to the HF group, however, we chose to examine the wrist-flexor muscles, which are not involved in locomotor activity and thus should represent a relatively untrained state in both groups, independent of habitual physical activity levels. This assumption has been made in previous studies (Park et al. 1988; Ryan et al. 2013a). Overall, the results of our in vivo assessment showed a reduction in oxidative capacity independent of physical activity, suggesting the presence of a small but statistically significant impairment skeletal muscle oxidative capacity. The magnitude of impairment in oxidative capacity we detected was smaller than reported in previous studies (Drexler et al. 1992b; Mancini et al. 1992), but this was most likely the result of assessing oxidative capacity in an inactive muscle group. It remains to be seen if this level of dysfunction in oxidative metabolism is clinically relevant.

To further investigate the reduced oxidative capacity found in HF participants, we explored whether these participants would also have impaired oxidative adaptations in response to endurance training. A small subset of HF and control participants from the cross-sectional study agreed to participate in 4 weeks of wrist-flexor endurance exercise training. Following the exercise training, the participants with HF showed no improvement in oxidative capacity when compared to controls, despite matching the controls in progression of training volume (Fig.1). No significant fluctuations from baseline were observed at any point during the training, with the end-training result being a −2% change from baseline. Interestingly, these results do not agree with previous studies that have shown oxidative improvements to endurance exercise (Minotti et al. 1990; Hambrecht et al. 1995; Belardinelli et al. 1999). The discrepancy between this study and previous findings (Minotti et al. 1990; Hambrecht et al. 1995; Belardinelli et al. 1999) does not appear to be due to differences in HF populations between studies, as both physical (e.g., age and sex) and clinical (e.g., ejection fraction, type of HF) characteristics of the HF participants were similar. A potential explanation for this discrepancy could be found in pharmacotherapy. HF participants often take a wide array of medications, some of which are related to heart dysfunction (e.g., ACE inhibitors, digoxin, diuretics). Moreover, individuals with HF are also commonly prescribed medications for the control and treatment of comorbidities such as hypertension, diabetes, and hypercholesterolemia. Statins are used to treat hypercholesterolemia and have been shown to be myotoxic (Sinzinger and O'Grady 2004; Paiva et al. 2005). In fact, a recent study by Mikus et al. (2013) reported statin-induced attenuations in oxidative adaptations in overweight and sedentary subjects following 12-weeks of treadmill walking/jogging, a result which is strikingly similar to that observed in our population of patient with HF. Statin therapy has become increasingly widespread over the past several decades in the United States and in HF populations (Raina et al. 2006; National Center for Health Statistics 2011) because it is extremely effective at lowering cholesterol levels. Thus, it was not surprising that in the present study 14 out of the 16 HF participants in the cross-sectional study and six of the seven HF participants in the training study were taking statins. Since the previously discussed training studies (Minotti et al. 1990; Hambrecht et al. 1995; Belardinelli et al. 1999) did not report statin use, it is difficult to make comparisons, but the reduced oxidative capacity and lack of oxidative adaptations in response to exercise training in the HF group could have been caused by statin use. Moreover, as six of the seven HF patients in the exercise training cohort were taking statins, we were not able to distinguish the influence of HF from the possible influence of pharmacotherapy on the lack of oxidative adaptations. Unfortunately, this might be a difficult task for future studies to address as many people with HF are prescribed potentially myotoxic pharmacotherapies.

No differences were found in the resting mVO_2_ between HF and controls. Similarly, no differences in blood flow were detected between HF and controls. While the blood flow findings are in agreement with some previous research (LeJemtel et al. 1986; Tousoulis et al. 2005), Zelis et al. (1974) found forearm blood flow at rest was reduced by ∼43% in participants with HF.

There are several possible limitations with this study. The controls recruited in this study were a sample of convenience, thus several discrepancies existed between the HF and control group characteristics. The largest discrepancy was that HF group was composed of 80% males compared to only ∼25% males in the control group. We did not see a trend for sex differences in muscle oxidative capacity in our study, consistent with previous research (Kent-Braun and Ng 2000; Thompson et al. 2013). Moreover, all the females enrolled in this study were postmenopausal, which eliminated potential estrogen mediated differences in oxidative capacity (Capllonch-Amer et al. 2014; Cavalcanti-de-Albuquerque et al. 2014). A moderate difference in weight between the HF and control groups was found, but this was most likely due to the sex difference highlighted earlier. The second part of our study, which involved 1 month of forearm exercise training, had a relatively small sample size, as we found recruitment for a forearm only training study was difficult in this population. Future studies with larger sample sizes will be needed to confirm the finding of the training portion of our study. The training findings suggest that the impaired muscle oxidative capacity may be related to the cumulative effects of chronically impaired mitochondrial biogenesis rather than impaired mitochondrial function. The reductions in skeletal muscle oxidative capacity could be attributed to two primary alterations, (1) reduction in mitochondrial number; and (2) reduction in electron transport system function. A strength of the NIRS method is that the entire system is assessed in its native environment, but this might be considered a limitation, as we cannot distinguish between functional changes derived from mitochondrial number or electron transport system function. Future studies should seek to reproduce these findings, perhaps in more clinically relevant muscles such as the quadriceps, and elucidate the mechanisms behind the impairments in skeletal muscle oxidative capacity.

In summary, this study found reduced skeletal muscle oxidative capacity in a nonlocomotor muscle in participants with HF. This finding indicates that deconditioning may not be entirely responsible for reductions in oxidative muscle metabolism in HF. This study also found evidence suggesting that HF, in combination with the wide array of pharmacological therapies prescribed to treat both HF and frequent comorbidities associated with HF, may interfere with skeletal muscle oxidative adaptations to endurance exercise training. Our results provide clear evidence supporting dysfunctional oxidative metabolism in participants with HF. Future studies should be performed to elucidate the mechanism of impaired exercise adaptations, as well as, to investigate the potential clinical impact of reduced oxidative metabolism.

## Conflict of Interest

None declared.
